# 1,2-Bis(*N*′-benzoyl­thio­ureido)-4-chloro­benzene

**DOI:** 10.1107/S1600536811014954

**Published:** 2011-05-07

**Authors:** Bohari M. Yamin, Uwaisulqarni M. Osman

**Affiliations:** a School of Chemical Sciences and Food Technology, Universiti Kebangsaan Malaysia, UKM 43500 Bangi Selangor, Malaysia

## Abstract

In the title compound, C_22_H_17_ClN_4_O_2_S_2_, both benzoyl groups are *trans *to the thiono group across their C—N bonds. The two methyl­ene carbamothioyl formamide fragments of the benzoyl­thio­urea side arms make a dihedral angle of 87.00 (10)°. The mol­ecule is stabilized by intra­molecular N—H⋯O, N—H⋯S and C—H⋯·S hydrogen bonds. In the crystal, mol­ecules are linked by N—H⋯O and N—H⋯S inter­molecular hydrogen bonds into zigzag chains along the *a* axis.

## Related literature

For the structure of related bis­carbomothioyl thio­urea compounds, see: Thiam *et al.* (2008 ▶); Yusof *et al.* (2008 ▶); Woei Hung & Kassim (2010 ▶). For bond length data, see: Allen (2002 ▶).
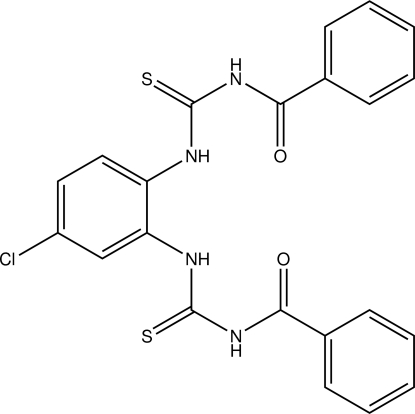


## Experimental

### 

#### Crystal data

C_22_H_17_ClN_4_O_2_S_2_
*M* *_r_* = 468.97
Triclinic, 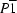

*a* = 9.637 (4) Å
*b* = 10.820 (4) Å
*c* = 11.370 (4) Å
α = 84.443 (8)°
β = 68.706 (8)°
γ = 86.551 (9)°
*V* = 1099.1 (7) Å^3^
*Z* = 2
Mo *K*α radiation
μ = 0.39 mm^−1^
*T* = 298 K
0.49 × 0.16 × 0.09 mm

#### Data collection

Bruker SMART APEX CCD area-detector diffractometer
Absorption correction: multi-scan (*SADABS*; Bruker, 2000 ▶) *T*_min_ = 0.928, *T*_max_ = 0.965
12132 measured reflections
4083 independent reflections
3107 reflections with *I* > 2σ(*I*)
*R*_int_ = 0.042

#### Refinement

*R*[*F* ^2^ > 2σ(*F* ^2^)] = 0.066
*wR*(*F* ^2^) = 0.173
*S* = 1.08
4083 reflections
280 parameters
H-atom parameters constrained
Δρ_max_ = 0.79 e Å^−3^
Δρ_min_ = −0.30 e Å^−3^

### 

Data collection: *SMART* (Bruker, 2000 ▶); cell refinement: *SAINT* (Bruker, 2000 ▶); data reduction: *SAINT* (Bruker, 2000 ▶); program(s) used to solve structure: *SHELXTL* (Sheldrick, 2008 ▶); program(s) used to refine structure: *SHELXTL* (Sheldrick, 2008 ▶); molecular graphics: *SHELXTL* (Sheldrick, 2008 ▶); software used to prepare material for publication: *SHELXTL* (Sheldrick, 2008 ▶), *PARST* (Nardelli, 1995 ▶) and *PLATON* (Spek, 2009 ▶).

## Supplementary Material

Crystal structure: contains datablocks global, I. DOI: 10.1107/S1600536811014954/ff2008sup1.cif

Structure factors: contains datablocks I. DOI: 10.1107/S1600536811014954/ff2008Isup2.hkl

Supplementary material file. DOI: 10.1107/S1600536811014954/ff2008Isup3.cml

Additional supplementary materials:  crystallographic information; 3D view; checkCIF report

## Figures and Tables

**Table 1:** Hydrogen-bond geometry (Å, °)

*D*—H⋯*A*	*D*—H	H⋯*A*	*D*⋯*A*	*D*—H⋯*A*
N2—H2*A*⋯S2	0.86	2.86	3.443 (4)	127
N2—H2*A*⋯O1	0.86	1.91	2.621 (5)	140
N3—H3*A*⋯O2	0.86	1.96	2.642 (3)	135
C10—H10*A*⋯S1	0.93	2.61	3.173 (5)	120
N1—H1*A*⋯O2^i^	0.86	2.51	3.328 (5)	159
N4—H4*A*⋯S2^ii^	0.86	2.81	3.476 (4)	136

Symmetry codes: (i) 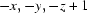; (ii) 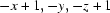.

**Fig. 1.**
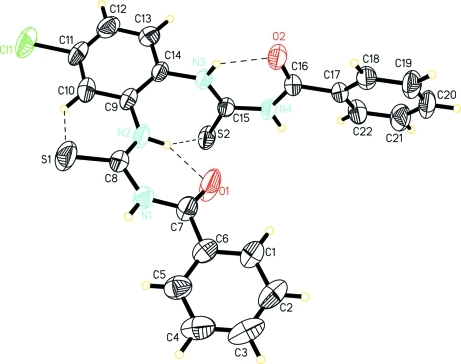
The molecular structure of the title compound, with displacement ellipsoids drawn at the 50% probability level.

**Fig. 2.**
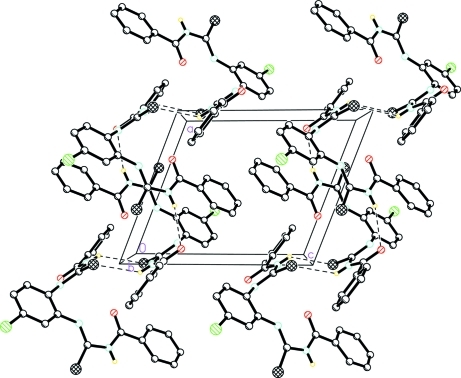
A packing diagram of the title compound viewed down the a-axis. Hydrogen bonds are shown by dashed lines.

## supplementary crystallographic information

### Comment

Bis-carbonoyl thiourea compounds are relatively less reported than their mono-carbonoyl thiourea derivatives. The title compound contains two benzoyl thioureido groups connected by 4-chlorobenzene bridge at 1, 2 position (Fig.1). The C7—O1 and C16—O2 bond lengths in the both side arms of 1.220 (5) and 1.230 (5) Å, respectively, are slightly longer than the normal C=O double bonds (1.200 Å) and comparable to those in 1,2-bis(*N*'-benzoylthioureido)benzene (Thiam *et al.* 2008) and 1-benzoyl-3-[4-(3-benzoylthioureido)-phenyl]thiourea (Woei Hung & Kassim 2010). Other bond lengths and angles are in normal ranges (Allen, 2002). Both methylene carbamothioyl formamide, S1/O1/N1/N2/C6/C7/C8/C9 and S2/O2/N3/N4/C14/C15/C16 fragments of the benzoyl thiourea side arms are planar with maximum deviation of 0.060 (3)Å for O1 atom and make dihedral angles of 87.00 (10)°. The dihedral angle between (C1—C6) and (C17—C22) benzene rings is 86.4 (2)° to each other. There are four intramolecular hydrogen bonds forming three pseudo-six-membered ring [S1···H10A—C10—C9—N2—C8], [O1···H2A—N2—C8—N1—C7] and [O2···H3A—N3—C15—N4—C16] and one pseudo-seven-membered ring [S2···H2A—N2—C9—C14—N3—C15] as compared to two intramolecular hydrogen bonds observed in 1,2-bis(*N*'-benzoylthioureido) benzene (Thiam *et al.* 2008) and 1,2-bis[*N*'-(2,2-dimethylpropionyl)thioureido] cyclohexane (Yusof *et al.* 2008). In the crystal structure, the molecules are linked by N1—H1A···O2 and N4—H4A···S2 intermolecular hydrogen bonds (symmetry codes as in Table 2) into a zigzag chains along the *a* axis.

### Experimental

To a stirring acetone solution (75 ml) of benzoyl chloride (0.04 mol) and ammonium thiocyanate (0.04 mol). 4-chlorobenzene-1,2-diamine (0.02 mol) in 40 ml of acetone was added dropwise. The solution mixture was refluxed for 1 h. The resulting solution was poured into a beaker containing some ice cubes. The white precipitate was filtered off and washed with distilled water and cold ethanol before dried under vacuum. Good quality crystals were obtained by recrystallization from ethanol.

### Refinement

H atoms on the parent carbon and nitrogen atoms were positioned geometrically with C—H= 0.93Å and N—H = 0.86 Å, constrained to ride on their parent atoms with *U*_iso_(H)=*xU*_eq_(parent atom) where *x*=1.2 for both CH and NH groups. There are highest peak of 0.88Å from H12A and deepest hole 0.91Å from S1.

### Crystal data

**Table:**

C_22_H_17_ClN_4_O_2_S_2_	*Z* = 2
*M**_r_* = 468.97	*F*(000) = 484
Triclinic, *P*1	*D*_x_ = 1.417 Mg m^−^^3^
Hall symbol: -P 1	Melting point = 458.5–459.5 K
*a* = 9.637 (4) Å	Mo *K*α radiation, λ = 0.71073 Å
*b* = 10.820 (4) Å	Cell parameters from 2481 reflections
*c* = 11.370 (4) Å	θ = 1.8–25.5°
α = 84.443 (8)°	µ = 0.39 mm^−^^1^
β = 68.706 (8)°	*T* = 298 K
γ = 86.551 (9)°	Plate, colourless
*V* = 1099.1 (7) Å^3^	0.49 × 0.16 × 0.09 mm

### Data collection

**Table:**

Bruker SMART APEX CCD area-detector diffractometer	4083 independent reflections
Radiation source: fine-focus sealed tube	3107 reflections with *I* > 2σ(*I*)
graphite	*R*_int_ = 0.042
Detector resolution: 83.66 pixels mm^-1^	θ_max_ = 25.5°, θ_min_ = 1.8°
ω scans	*h* = −11→11
Absorption correction: multi-scan (*SADABS*; Bruker, 2000)	*k* = −13→13
*T*_min_ = 0.928, *T*_max_ = 0.965	*l* = −13→13
12132 measured reflections	

### Refinement

**Table:**

Refinement on *F*^2^	Primary atom site location: structure-invariant direct methods
Least-squares matrix: full	Secondary atom site location: difference Fourier map
*R*[*F*^2^ > 2σ(*F*^2^)] = 0.066	Hydrogen site location: inferred from neighbouring sites
*wR*(*F*^2^) = 0.173	H-atom parameters constrained
*S* = 1.08	*w* = 1/[σ^2^(*F*_o_^2^) + (0.0812*P*)^2^ + 0.6319*P*] where *P* = (*F*_o_^2^ + 2*F*_c_^2^)/3
4083 reflections	(Δ/σ)_max_ < 0.001
280 parameters	Δρ_max_ = 0.79 e Å^−^^3^
0 restraints	Δρ_min_ = −0.30 e Å^−^^3^

### Special details

**Table:**

Geometry. All e.s.d.'s (except the e.s.d. in the dihedral angle between two l.s. planes) are estimated using the full covariance matrix. The cell e.s.d.'s are taken into account individually in the estimation of e.s.d.'s in distances, angles and torsion angles; correlations between e.s.d.'s in cell parameters are only used when they are defined by crystal symmetry. An approximate (isotropic) treatment of cell e.s.d.'s is used for estimating e.s.d.'s involving l.s. planes.
Refinement. Refinement of *F*^2^ against ALL reflections. The weighted *R*-factor *wR* and goodness of fit *S* are based on *F*^2^, conventional *R*-factors *R* are based on *F*, with *F* set to zero for negative *F*^2^. The threshold expression of *F*^2^ > σ(*F*^2^) is used only for calculating *R*-factors(gt) *etc*. and is not relevant to the choice of reflections for refinement. *R*-factors based on *F*^2^ are statistically about twice as large as those based on *F*, and *R*- factors based on ALL data will be even larger.

### Fractional atomic coordinates and isotropic or equivalent isotropic displacement parameters (Å^2^)

**Table:**

	*x*	*y*	*z*	*U*_iso_*/*U*_eq_	
Cl1	0.16623 (15)	0.60564 (10)	0.11914 (13)	0.0845 (4)	
S1	−0.13518 (13)	0.32671 (13)	0.50891 (12)	0.0882 (5)	
S2	0.50891 (11)	0.17508 (8)	0.38266 (10)	0.0524 (3)	
O1	0.1820 (3)	0.0277 (3)	0.5716 (3)	0.0866 (11)	
O2	0.3898 (3)	−0.1381 (2)	0.2029 (3)	0.0570 (7)	
N1	−0.0285 (3)	0.1455 (3)	0.6212 (3)	0.0504 (7)	
H1A	−0.1142	0.1557	0.6795	0.060*	
N2	0.1368 (3)	0.2180 (3)	0.4276 (3)	0.0486 (7)	
H2A	0.1939	0.1630	0.4482	0.058*	
N3	0.3678 (3)	0.1001 (2)	0.2425 (2)	0.0400 (6)	
H3A	0.3400	0.0403	0.2122	0.048*	
N4	0.4966 (3)	−0.0519 (2)	0.3218 (3)	0.0439 (7)	
H4A	0.5480	−0.0695	0.3695	0.053*	
C1	0.0621 (5)	−0.1393 (4)	0.7813 (4)	0.0604 (10)	
H1B	0.1408	−0.1673	0.7127	0.072*	
C2	0.0101 (6)	−0.2134 (4)	0.8932 (5)	0.0743 (13)	
H2B	0.0531	−0.2919	0.8994	0.089*	
C3	−0.1036 (6)	−0.1724 (5)	0.9943 (5)	0.0819 (14)	
H3B	−0.1391	−0.2234	1.0691	0.098*	
C4	−0.1669 (5)	−0.0552 (5)	0.9864 (4)	0.0759 (13)	
H4B	−0.2428	−0.0267	1.0567	0.091*	
C5	−0.1177 (4)	0.0195 (4)	0.8750 (4)	0.0577 (10)	
H5A	−0.1614	0.0978	0.8695	0.069*	
C6	−0.0031 (4)	−0.0226 (3)	0.7712 (3)	0.0496 (9)	
C7	0.0582 (4)	0.0508 (3)	0.6474 (3)	0.0497 (9)	
C8	0.0013 (4)	0.2288 (3)	0.5134 (3)	0.0466 (8)	
C9	0.2023 (4)	0.2824 (3)	0.3077 (3)	0.0415 (8)	
C10	0.1586 (4)	0.4028 (3)	0.2774 (4)	0.0526 (9)	
H10A	0.0848	0.4461	0.3378	0.063*	
C11	0.2265 (4)	0.4562 (3)	0.1565 (4)	0.0530 (9)	
C12	0.3378 (5)	0.3993 (3)	0.0662 (4)	0.0594 (10)	
H12A	0.3811	0.4381	−0.0147	0.071*	
C13	0.3855 (4)	0.2818 (3)	0.0974 (3)	0.0514 (9)	
H13A	0.4631	0.2415	0.0371	0.062*	
C14	0.3192 (4)	0.2239 (3)	0.2167 (3)	0.0394 (7)	
C15	0.4541 (3)	0.0725 (3)	0.3110 (3)	0.0393 (7)	
C16	0.4686 (4)	−0.1497 (3)	0.2677 (3)	0.0403 (7)	
C17	0.5404 (3)	−0.2712 (3)	0.2888 (3)	0.0392 (7)	
C22	0.6633 (4)	−0.2819 (3)	0.3250 (4)	0.0526 (9)	
H18A	0.7052	−0.2112	0.3372	0.063*	
C21	0.7232 (5)	−0.3984 (4)	0.3429 (4)	0.0673 (11)	
H19A	0.8056	−0.4062	0.3675	0.081*	
C20	0.6622 (5)	−0.5012 (4)	0.3248 (4)	0.0693 (12)	
H20A	0.7035	−0.5791	0.3370	0.083*	
C19	0.5408 (5)	−0.4921 (3)	0.2890 (4)	0.0642 (11)	
H21A	0.4997	−0.5634	0.2773	0.077*	
C18	0.4797 (4)	−0.3769 (3)	0.2702 (3)	0.0488 (8)	
H22A	0.3978	−0.3702	0.2450	0.059*	

### Atomic displacement parameters (Å^2^)

**Table:**

	*U*^11^	*U*^22^	*U*^33^	*U*^12^	*U*^13^	*U*^23^
Cl1	0.1099 (10)	0.0483 (6)	0.0988 (9)	0.0119 (6)	−0.0510 (7)	0.0225 (6)
S1	0.0625 (7)	0.1060 (10)	0.0712 (7)	0.0420 (7)	−0.0088 (6)	0.0230 (7)
S2	0.0667 (6)	0.0323 (4)	0.0726 (6)	0.0039 (4)	−0.0429 (5)	−0.0049 (4)
O1	0.0605 (18)	0.0737 (19)	0.087 (2)	0.0224 (15)	0.0032 (16)	0.0421 (16)
O2	0.0740 (17)	0.0382 (13)	0.0780 (18)	0.0112 (12)	−0.0508 (15)	−0.0109 (12)
N1	0.0401 (15)	0.0577 (18)	0.0452 (16)	0.0099 (13)	−0.0098 (13)	0.0052 (14)
N2	0.0436 (16)	0.0429 (16)	0.0534 (17)	0.0123 (12)	−0.0160 (14)	0.0093 (13)
N3	0.0491 (16)	0.0318 (13)	0.0444 (15)	0.0029 (11)	−0.0241 (13)	−0.0024 (11)
N4	0.0546 (17)	0.0326 (14)	0.0528 (17)	0.0124 (12)	−0.0305 (14)	−0.0064 (12)
C1	0.066 (2)	0.054 (2)	0.065 (2)	−0.0002 (18)	−0.030 (2)	0.0100 (19)
C2	0.091 (3)	0.063 (3)	0.076 (3)	−0.015 (2)	−0.044 (3)	0.026 (2)
C3	0.089 (3)	0.101 (4)	0.060 (3)	−0.031 (3)	−0.038 (3)	0.032 (3)
C4	0.063 (3)	0.112 (4)	0.049 (2)	−0.011 (3)	−0.019 (2)	0.006 (2)
C5	0.051 (2)	0.075 (3)	0.051 (2)	−0.0052 (19)	−0.0231 (18)	0.0013 (19)
C6	0.046 (2)	0.054 (2)	0.054 (2)	−0.0110 (16)	−0.0269 (18)	0.0067 (17)
C7	0.045 (2)	0.046 (2)	0.055 (2)	0.0010 (15)	−0.0170 (17)	0.0080 (16)
C8	0.050 (2)	0.0447 (19)	0.0452 (19)	0.0094 (15)	−0.0195 (16)	−0.0017 (15)
C9	0.0456 (19)	0.0334 (16)	0.0503 (19)	0.0009 (14)	−0.0255 (16)	0.0061 (14)
C10	0.055 (2)	0.0405 (19)	0.064 (2)	0.0103 (16)	−0.0271 (19)	0.0000 (17)
C11	0.066 (2)	0.0375 (18)	0.063 (2)	−0.0026 (17)	−0.037 (2)	0.0152 (17)
C12	0.079 (3)	0.049 (2)	0.051 (2)	−0.0070 (19)	−0.028 (2)	0.0135 (18)
C13	0.064 (2)	0.047 (2)	0.043 (2)	0.0006 (17)	−0.0201 (17)	0.0017 (16)
C14	0.0483 (19)	0.0310 (15)	0.0449 (18)	0.0024 (14)	−0.0254 (15)	0.0014 (14)
C15	0.0402 (17)	0.0361 (17)	0.0398 (17)	0.0047 (13)	−0.0139 (14)	−0.0004 (13)
C16	0.0415 (18)	0.0323 (16)	0.0465 (18)	0.0039 (13)	−0.0158 (15)	−0.0037 (14)
C17	0.0429 (18)	0.0331 (16)	0.0410 (17)	0.0066 (13)	−0.0148 (14)	−0.0059 (13)
C22	0.052 (2)	0.0411 (19)	0.069 (2)	0.0076 (16)	−0.0261 (19)	−0.0121 (17)
C21	0.064 (3)	0.060 (2)	0.087 (3)	0.024 (2)	−0.040 (2)	−0.010 (2)
C20	0.094 (3)	0.036 (2)	0.085 (3)	0.020 (2)	−0.045 (3)	−0.0045 (19)
C19	0.086 (3)	0.0315 (18)	0.081 (3)	0.0004 (18)	−0.039 (2)	−0.0004 (18)
C18	0.053 (2)	0.0397 (18)	0.055 (2)	0.0037 (15)	−0.0214 (17)	−0.0054 (15)

### Geometric parameters (Å, °)

**Table:**

Cl1—C11	1.750 (3)	C4—H4B	0.9300
S1—C8	1.651 (3)	C5—C6	1.385 (5)
S2—C15	1.653 (3)	C5—H5A	0.9300
O1—C7	1.220 (4)	C6—C7	1.482 (5)
O2—C16	1.229 (4)	C9—C14	1.391 (5)
N1—C7	1.363 (4)	C9—C10	1.395 (4)
N1—C8	1.399 (4)	C10—C11	1.375 (5)
N1—H1A	0.8600	C10—H10A	0.9300
N2—C8	1.322 (4)	C11—C12	1.351 (6)
N2—C9	1.409 (4)	C12—C13	1.384 (5)
N2—H2A	0.8600	C12—H12A	0.9300
N3—C15	1.335 (4)	C13—C14	1.376 (5)
N3—C14	1.431 (4)	C13—H13A	0.9300
N3—H3A	0.8600	C16—C17	1.488 (4)
N4—C16	1.361 (4)	C17—C18	1.382 (5)
N4—C15	1.390 (4)	C17—C22	1.384 (5)
N4—H4A	0.8600	C22—C21	1.383 (5)
C1—C2	1.379 (6)	C22—H18A	0.9300
C1—C6	1.390 (5)	C21—C20	1.355 (6)
C1—H1B	0.9300	C21—H19A	0.9300
C2—C3	1.358 (7)	C20—C19	1.367 (6)
C2—H2B	0.9300	C20—H20A	0.9300
C3—C4	1.383 (7)	C19—C18	1.377 (5)
C3—H3B	0.9300	C19—H21A	0.9300
C4—C5	1.376 (6)	C18—H22A	0.9300
			
C7—N1—C8	129.6 (3)	C11—C10—C9	118.8 (3)
C7—N1—H1A	115.2	C11—C10—H10A	120.6
C8—N1—H1A	115.2	C9—C10—H10A	120.6
C8—N2—C9	130.2 (3)	C12—C11—C10	123.1 (3)
C8—N2—H2A	114.9	C12—C11—Cl1	118.8 (3)
C9—N2—H2A	114.9	C10—C11—Cl1	118.0 (3)
C15—N3—C14	123.5 (3)	C11—C12—C13	118.3 (3)
C15—N3—H3A	118.2	C11—C12—H12A	120.9
C14—N3—H3A	118.2	C13—C12—H12A	120.9
C16—N4—C15	129.3 (3)	C14—C13—C12	120.6 (3)
C16—N4—H4A	115.4	C14—C13—H13A	119.7
C15—N4—H4A	115.4	C12—C13—H13A	119.7
C2—C1—C6	119.9 (4)	C13—C14—C9	120.5 (3)
C2—C1—H1B	120.0	C13—C14—N3	118.5 (3)
C6—C1—H1B	120.0	C9—C14—N3	120.9 (3)
C3—C2—C1	120.3 (4)	N3—C15—N4	115.9 (3)
C3—C2—H2B	119.8	N3—C15—S2	124.3 (2)
C1—C2—H2B	119.8	N4—C15—S2	119.8 (2)
C2—C3—C4	120.2 (4)	O2—C16—N4	121.8 (3)
C2—C3—H3B	119.9	O2—C16—C17	121.5 (3)
C4—C3—H3B	119.9	N4—C16—C17	116.7 (3)
C5—C4—C3	120.3 (4)	C18—C17—C22	119.6 (3)
C5—C4—H4B	119.9	C18—C17—C16	117.3 (3)
C3—C4—H4B	119.9	C22—C17—C16	123.1 (3)
C4—C5—C6	119.7 (4)	C21—C22—C17	119.4 (3)
C4—C5—H5A	120.2	C21—C22—H18A	120.3
C6—C5—H5A	120.2	C17—C22—H18A	120.3
C5—C6—C1	119.5 (4)	C20—C21—C22	120.3 (4)
C5—C6—C7	123.8 (3)	C20—C21—H19A	119.9
C1—C6—C7	116.6 (3)	C22—C21—H19A	119.9
O1—C7—N1	121.0 (3)	C21—C20—C19	120.9 (3)
O1—C7—C6	121.4 (3)	C21—C20—H20A	119.5
N1—C7—C6	117.6 (3)	C19—C20—H20A	119.5
N2—C8—N1	114.9 (3)	C20—C19—C18	119.7 (4)
N2—C8—S1	128.4 (3)	C20—C19—H21A	120.1
N1—C8—S1	116.7 (3)	C18—C19—H21A	120.1
C14—C9—C10	118.6 (3)	C19—C18—C17	120.0 (3)
C14—C9—N2	118.1 (3)	C19—C18—H22A	120.0
C10—C9—N2	123.2 (3)	C17—C18—H22A	120.0
			
C6—C1—C2—C3	−0.8 (6)	C12—C13—C14—C9	0.4 (5)
C1—C2—C3—C4	−0.9 (7)	C12—C13—C14—N3	177.1 (3)
C2—C3—C4—C5	1.8 (7)	C10—C9—C14—C13	−2.9 (5)
C3—C4—C5—C6	−1.0 (6)	N2—C9—C14—C13	178.9 (3)
C4—C5—C6—C1	−0.7 (5)	C10—C9—C14—N3	−179.5 (3)
C4—C5—C6—C7	−179.7 (3)	N2—C9—C14—N3	2.2 (4)
C2—C1—C6—C5	1.6 (6)	C15—N3—C14—C13	105.1 (4)
C2—C1—C6—C7	−179.4 (4)	C15—N3—C14—C9	−78.2 (4)
C8—N1—C7—O1	0.0 (6)	C14—N3—C15—N4	−177.1 (3)
C8—N1—C7—C6	179.8 (3)	C14—N3—C15—S2	3.7 (4)
C5—C6—C7—O1	159.2 (4)	C16—N4—C15—N3	3.3 (5)
C1—C6—C7—O1	−19.8 (5)	C16—N4—C15—S2	−177.5 (3)
C5—C6—C7—N1	−20.6 (5)	C15—N4—C16—O2	−3.9 (5)
C1—C6—C7—N1	160.4 (3)	C15—N4—C16—C17	175.0 (3)
C9—N2—C8—N1	177.6 (3)	O2—C16—C17—C18	−20.7 (5)
C9—N2—C8—S1	−2.3 (6)	N4—C16—C17—C18	160.4 (3)
C7—N1—C8—N2	−4.2 (5)	O2—C16—C17—C22	159.0 (3)
C7—N1—C8—S1	175.7 (3)	N4—C16—C17—C22	−19.8 (5)
C8—N2—C9—C14	−154.5 (3)	C18—C17—C22—C21	−0.4 (5)
C8—N2—C9—C10	27.3 (6)	C16—C17—C22—C21	179.9 (3)
C14—C9—C10—C11	3.8 (5)	C17—C22—C21—C20	0.2 (6)
N2—C9—C10—C11	−178.1 (3)	C22—C21—C20—C19	−0.1 (7)
C9—C10—C11—C12	−2.4 (6)	C21—C20—C19—C18	0.3 (7)
C9—C10—C11—Cl1	178.2 (3)	C20—C19—C18—C17	−0.6 (6)
C10—C11—C12—C13	−0.1 (6)	C22—C17—C18—C19	0.6 (5)
Cl1—C11—C12—C13	179.3 (3)	C16—C17—C18—C19	−179.6 (3)
C11—C12—C13—C14	1.1 (6)		

### Hydrogen-bond geometry (Å, °)

**Table:**

*D*—H···*A*	*D*—H	H···*A*	*D*···*A*	*D*—H···*A*
N2—H2A···S2	0.86	2.86	3.443 (4)	127
N2—H2A···O1	0.86	1.91	2.621 (5)	140
N2—H2A···N3	0.86	2.46	2.791 (4)	103
N3—H3A···O2	0.86	1.96	2.642 (3)	135
C10—H10A···S1	0.93	2.61	3.173 (5)	120
N1—H1A···O2^i^	0.86	2.51	3.328 (5)	159
N4—H4A···S2^ii^	0.86	2.81	3.476 (4)	136

Symmetry codes:  (i) −*x*, −*y*, −*z*+1; (ii) −*x*+1, −*y*, −*z*+1.
